# AtUBL5 regulates growth and development through pre-mRNA splicing in *Arabidopsis thaliana*

**DOI:** 10.1371/journal.pone.0224795

**Published:** 2019-11-15

**Authors:** Etsuko Watanabe, Shoji Mano, Mikio Nishimura, Kenji Yamada

**Affiliations:** 1 Department of Cell Biology, National Institute for Basic Biology, Okazaki, Japan; 2 Department of Basic Biology, School of Life Science, SOKENDAI (The Graduate University for Advanced Studies), Okazaki, Japan; 3 Department of Biology, Faculty of Science and Engineering, Konan University, Kobe, Japan; 4 Malopolska Center of Biotechnology, Jagiellonian University, Krakow, Poland; International Centre for Genetic Engineering and Biotechnology, ITALY

## Abstract

Ubiquitin-like proteins play important roles in the regulation of many biological processes. UBL5 (Ubiquitin-like protein 5)/Hub1 (Homologous to ubiquitin 1), a member of the ubiquitin family, acts as a ubiquitin-like modifier on a specific target, the spliceosomal protein Snu66, in yeast and human cells. The 22^nd^ aspartic acid (Asp22) is involved in the attachment of Hub1 to the Hub1 interaction domain (HIND) of Snu66 in yeast to modulate spliceosomal activity. Hub1 differs from other modifiers which interact covalently with their targets. It modulates pre-mRNA splicing by binding to Snu66 non-covalently in both yeast and human cells. However, the molecular mechanisms of Hub1-mediated pre-mRNA splicing in plant systems remains unclear. To better understand the function of Hub1 in plants, we examined the role of this ubiquitin-like modifier in *Arabidopsis thaliana*, which has two Hub1 homologues. *Arabidopsis* UBL5/Hub1(UBL5) is highly conserved at the amino acid level, compared to eukaryotic homologues in both plants and animals. In this study, phenotypic analysis of *A*. *thaliana* with reduced *UBL5* gene expression, generated by RNA interference of *AtUBL5a* and *AtUBL5b* were performed. Interestingly, knock down plants of AtUBL5 showed abnormalities in root elongation, plant development, and auxin response. *AtUBL5b* is highly expressed in the vascular tissue of the leaf, stem, and root tissue. Yeast two-hybrid analysis revealed that AtUBL5a and AtUBL5b interact with the putative splicing factor AtPRP38 through its C-terminal domain (AtPRP38C). Knock down of *AtUBL5b* resulted in a pattern of insufficient pre-mRNA splicing in several introns of *AtCDC2*, and in introns of *IAA1*, *IAA4*, and *IAA5*. Defects of pre-mRNA splicing in an *AtPRP38* mutant resulted in an insufficient pre-mRNA splicing pattern in the intron of *IAA1*. Based on these results, we showed that AtUBL5b positively regulates plant root elongation and development through pre-mRNA splicing with AtPRP38C in *A*. *thaliana*.

## Introduction

Ubiquitin and ubiquitin-like proteins (UBLs) are multifunctional regulatory modifiers of eukaryotic and eubacterial proteins [1]. Research on yeast and human homologous to ubiquitin 1 (Hub1, also known as Ubl5) protein led to the discovery of Hub1-dependent splicing processes [2–7]. Conditional *Schizosaccharomyces pombe* mutants containing a temperature-sensitive *Hub1* allele display pre-mRNA splicing defects at high temperatures [6]. Misha et al observed unusual splicing of 5′ splicing sites (5′-ss) at noncanonical sequences in *Saccharomyces cerevisiae*. They found that splicing of the highly conserved 5′-ss (GUAUGU) proceeds in a Hub1-independent manner, but splicing of the alternative 5′-ss sequences occurs in a Hub1-dependent manner [5]. Hub1 binding to *S*. *cerevisiae* Snu66 (ScSnu66) affects the interaction with spliceosomal proteins and splicing [5]. Spliceosomes that lack Hub1 cannot recognize noncanonical 5′-ss and are defective in alternative splicing of *Steroid receptor coactivator 1* (*SRC1)* in *S*. *cerevisiae* [5, 6]. In most organisms, pre-mRNA processing at noncanonical splice sites results in intron retention, exon skipping, or alternative splicing products. However, because of the functions of Hub1 and ScSnu66, the unusual pre-mRNA processing events are maintained at a low rate. Hub1 also binds to the DEAD-box helicase Pre-mRNA Processing 5 (Prp5), a key regulator of early spliceosome assembly, through a second functional interaction surface on Hub1, which is different from the Hub1 interaction domain (HIND) [3]. The interaction with Hub1 stimulates the ATPase activity of Prp5, which results in improved splicing efficiency, but it also decreases splicing fidelity and increases mis-splicing [3]. Knockout of Hub1 causes reduction in pre-mRNA splicing, and depletion of Hub1 reduces viability in human cells [8]. Hub1 depletion also elicits phenotypic abnormalities such as splicing speckle and mitotic defects, culminating in caspase-mediated apoptosis [2]. Hub1 has important functions as a modulator of spliceosome activity and a facilitator for alternative splicing in both animal and yeast cells [2]. In *Caenorhabditis elegans*, Hub1 was identified by screening for genes implicated in the unfolded protein response in mitochondria [9]. Immunoprecipitation analysis indicated that *C*. *elegans* and mammalian Hub1 associate with the DVE-1 transcription factor, which is responsible for initiating the unfolded protein response pathway in mitochondria [10]. Hub1 would therefore be involved during the stress response to process pre-mRNA into mRNA. Hub1 is a small ubiquitin-like protein that has ~20% sequence identity with ubiquitin, and is homologous to other ubiquitin family proteins, including SUMO and RUB [4, 6, 11–13]. Hub1 does not have the common C-terminal di-glycine motif, which is conserved among ubiquitin-like proteins and binds covalently to an amino group in their target proteins [1, 12, 13]. The C-terminal domain of Hub1 is not important for target binding; rather, it is the 22^nd^ aspartic acid (Asp22) that is involved in the attachment of Hub1 to the HIND of Snu66 in yeast and human cells [2, 4–7]. Therefore, although Hub1 is homologous to ubiquitin-like modifiers, it acts via a unique mechanism and represents a distinctive functional ubiquitin-like modifier system.

In contrast with the relatively well-known functions of Hub1 in animals and yeast, the biological role of Hub1 in plants remains unclear. Two genes, *AtUBL5a* and *AtUBL5b*, are identified in the genome of *Arabidopsis thaliana* and have amino acid sequences with high similarity to Hub1 proteins in yeast and humans. Intriguingly, in *A*. *thaliana*, Snu66 does not have a HIND, while AtPRP38, which is another component of the spliceosome complex, possesses a HIND in its C-terminal region. In perennial ryegrass (*Lolium perenne* L.), overexpression of LpHub1 improves drought tolerance [14]. Although the exact mechanism has not been experimentally assessed, improved stress tolerance in plants overexpressing LpHub1 was attributed to the regulation of signaling pathways associated with the stress response [14].

Auxin plays several important roles in the stress response, and plant growth and development, such as primary root elongation and lateral root development [15]. Transport inhibitor response 1/Auxin related F-box (TIR1/AFB) proteins are auxin receptors that are composed of a ubiquitin protein ligase E3 complex called SCF, which directs auxin/indole 3-acetic acid (Aux/IAA) to the ubiquitin-dependent degradation pathway [16, 17]. Aux/IAA genes are expressed early in the auxin response, and encode short-lived nuclear proteins that regulate the auxin response in higher plants.

To examine the mechanism of action and the biological function of UBL5 in higher plants, we used *Arabidopsis* RNAi to knockdown the gene expression of *AtUBL5a* and *AtUBL5b*. We evaluated the effect of AtUBL5 knockdown on plant development and response to auxin. We investigated the interaction of AtUBL5b with a HIND containing protein, the C-terminal region of AtPRP38, using two-hybrid systems. Finally, we assessed pre-mRNA splicing defects in *AtCDC2* and *IAA* genes in plants with suppressed *AtUBL5b* expression. In the present study, we demonstrated that AtUBL5 interacts with the HIND of AtPRP38C, and regulates root elongation and development by modulating pre-mRNA splicing in *AtUBL5* knockdown *A*. *thaliana*. AtUBL5 might work as a positive regulator of root elongation and development by regulating pre-mRNA splicing together with AtPRP38.

## Results and discussion

### Identification and subcellular localization of *A*. *thaliana* UBL5

**Fig 1:**
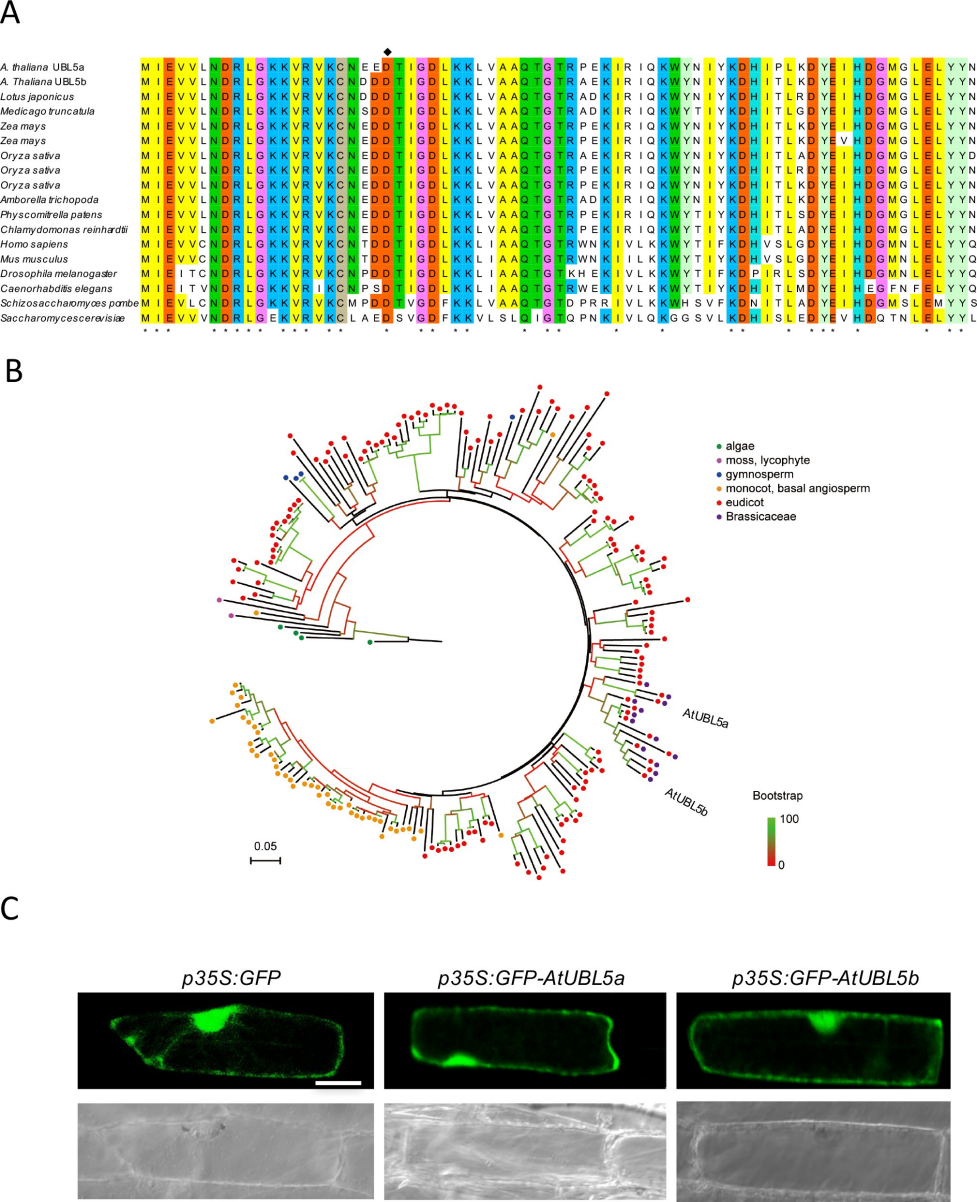
*A*. *thaliana* has two Hub1 homologues localized to the nucleus and cytosol. (A) Multiple protein sequence alignment of Hub1 from *Arabidopsis thaliana* (NP_190104.1, UBL5a; NP_199045.1, UBL5b), *Lotus japonicus* (AFK38458.1), *Medicago truncatula* (XP_003601666.1), *Zea mays* (NP_001147132.1, upper; NP_001341916.1, lower), *Oryza sativa* (BAS79881.1/Os02g0628800, upper; BAT15859.1/Os12g0143100, middle; BAT12654.1/Os11g0145400, lower), *Amborella trichopoda* (XP_006838813.1), *Physcomitrella patens* (XP_024369273.1), *Chlamydomonas reinhardtii* (XP_001697239.1), *Homo sapiens* (NP_077268.1), *Mus musculus* (NP_001159534.1), *Drosophila melanogaster* (NP_610239.1), *Caenorhabditis elegans* (NP_491640.1), *Schizosaccharomyces pombe* (NP_595099.1), and *Saccharomyces cerevisiae* (AAS56885.1). Conserved amino acids are highlighted as follows: A, M, I, V, and L are highlighted in yellow, E and D in orange, N, T, Q, W in green, R and K in blue, C in gray, G in pink, Y in light green, and H in light blue. The diamond indicates Asp22, which is involved in Hub1 binding to target proteins in yeast and humans. Asterisks indicate identical amino acids in Hub1 homologue sequences. (B) Phylogenetic tree of plant Hub1 protein coding regions in different species. Each filled circle indicates a Hub1 homologue. Circle colors denote the taxa in which Hub1 was identified. Stem colors represent bootstrap value. The scale denotes the ratio of base substitution per site. (C) Subcellular localization analysis of transient expression of GFP fusion protein with AtUBL5a and AtUBL5b in onion epidermal cells. Fluorescence microscopic images are shown in the upper row. The lower row shows the differential interference contrast (DIC) microscopy images. Bar = 50 μm.

There are two yeast Hub1 homologues in the genome of *A*. *thaliana* genome, which are designated as *AtUBL5a* (At3g45180) and *AtUBL5b* (At5g42300) (Fig 1A). Using database searching, we identified yeast Hub1 homologues in several model organisms. *Oryza sativa* and *Zea mays* have three and two Hub1 homologues in their genomes, respectively, whereas other model plants, animals, and yeast have a single Hub1 homologue in their genomes (Fig 1A). As a result of duplication of Hub1, plant Hub1 might have different roles or redundancy in model organisms like *O*. *sativa*. Each *Arabidopsis* Hub1 homologue might be involved in different biological processes in higher plants. Hub1 is essential for cell viability in yeast [6], but human cells with defective Hub1 are still viable [2]. There might be alternative pathways to compensate for defects in Hub1 in human cells. The amino acid identity was very high among Hub1 proteins from different organisms, indicating that AtUBL5 has a highly conserved role in these model systems. AtUBL5a and AtUBL5b share 94% amino acid sequence identity, and both are highly homologous to other plant UBL5 sequences. Both AtUBL5a and AtUBL5b amino acid sequences have an identity of 79% with human Hub1. Lysine (K) residues (Fig 1, highlighted in blue) are highly conserved in several positions in the Hub1 amino acid sequence, and are necessary to assemble polyubiquitin chains [1]. Although there is no direct evidence, these residues might be involved in ubiquitination for further modification of Hub1 [18]. However, lys12 and lys28 are conserved between *Arabidopsis* Ubiquitin-8 and AtUBL5a/b. Lys48 usually functions as a scaffold for polyubiquitin, but lysine 47 is present in AtUBL5, suggesting that further research is required [18]. These residues imply the ubiquitin-specific ability to bind to Hub1 or ubiquitin. Asp22 is also highly conserved among the analyzed organisms. In Hub1, this residue interacts with the HIND domain of target proteins (diamond in Fig 1A).

A phylogenic tree was created using the amino acid sequences of Hub1 homologues based on published plant genomes (Fig 1B). The generated phylogenic tree depicts the evolutionary time course of Hub1 in plant: algae and moss are close to the root, then gymnosperms and monocotyledons evolved, and finally eudicots separated from monocots. Hub1 protein in animals belongs to a different clade from plant Hub1 homologues (Fig 1B), suggesting that AtUBL51 might have a conserved role that is specific to green plants. The presence of three Hub1 homologues in the genome of *O*. *sativa* could be explained by its genome size, which is larger than that of *A*. *thaliana*. When the genome of *O*. *sativa* duplicated, the number of Hub1 homologues may have also increased. The study of diverse Hub1 homologues, like those in *O*. *sativa*, might reveal novel roles of this ubiquitin-like modifier.

To better understand the subcellular localization of AtUBL5a and AtUBL5b, we expressed these proteins transiently under a 35S promotor using the particle bombardment method in onion cells. The fluorescence from GFP-fused AtUBL5a or AtUBL5b was observed in both the nucleus and cytosol (Fig 1C). In *S*. *pombe*, Hub1 is also localized in the nucleus and cytosol [6]. However, AtUBL5a and AtUBL5b might have different functions in both nucleus and cytosol compared to *S*. *pombe*.

### RNAiUBL5b results in plant developmental defects

**Fig 2:**
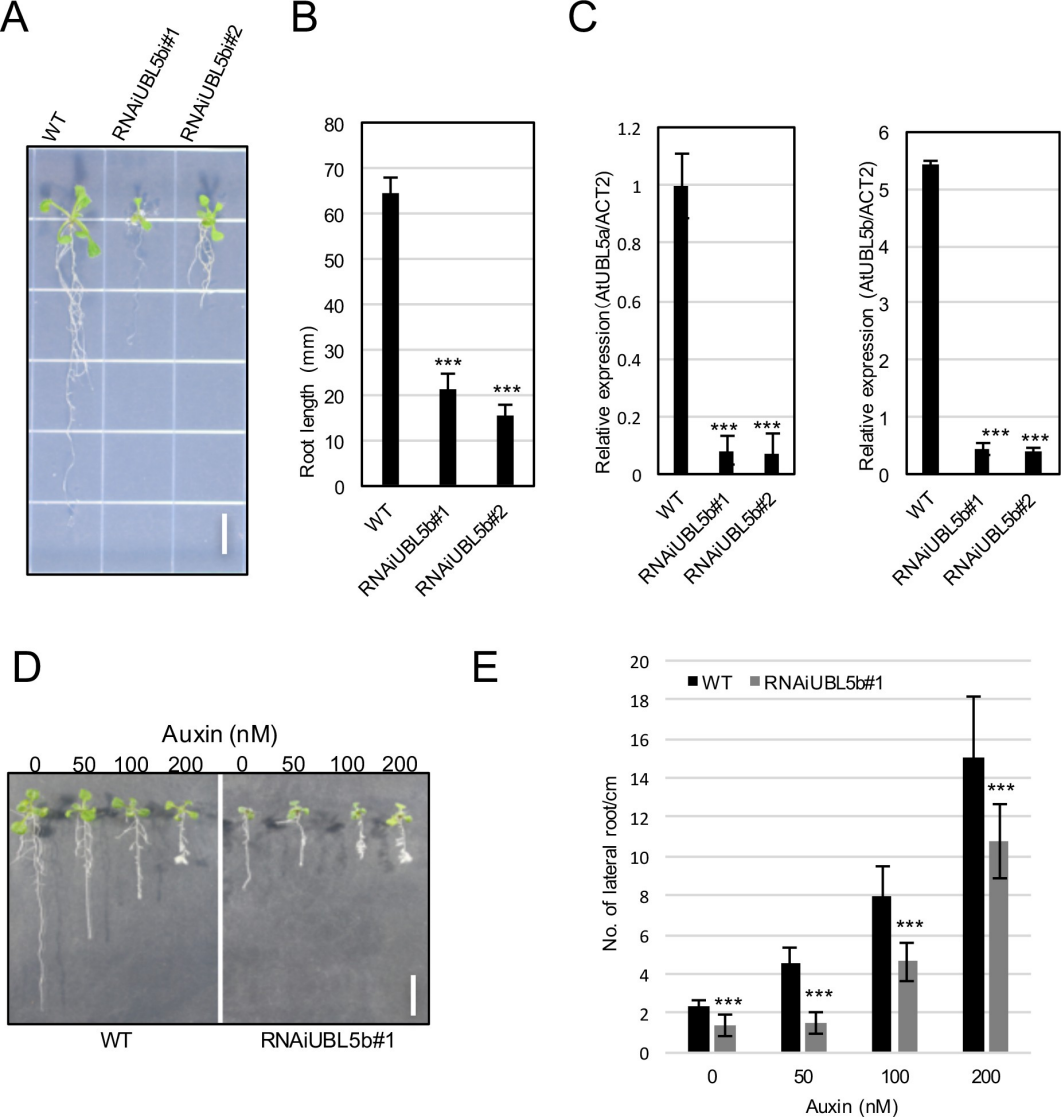
Phenotypic analysis of RNAiUBL5b in root elongation and development. (A) Growth of RNAiUBL5b#1, RNAiUBL5b#2, and WT. Plants were grown for 7 days. Bar = 5 mm. (B) Root lengths of RNAiUBL5b#1 and RNAiUBL5b#2 plants. (C) Relative expression levels of *AtUBL5a* and *AtUBL5b* in RNAiUBL5b#1 and RNAiUBL5b#2 plants were estimated by quantitative real time polymerase chain reaction (qRT-PCR) and compared to that of WT plants. The expression level of each gene was normalized to that of ACTIN 2 (*ACT2*). (D) Auxin sensitivity of RNAiUBL5b#1. RNAiUBL5b#1 and control WT seedlings were cultivated for 5 days, then transferred to MS media containing different concentrations of 2,4-D (auxin). Bar = 10 mm. (E) The sensitivity of RNAiUBL5b#1 and WT to auxin was assessed by measuring root elongation defects and the number of lateral roots. Values in (B), (C), and (E) represent mean ± standard deviations of 8–10 samples of three independent experiments. Asterisks in (B), (C), and (E) indicate statistical significance according to Student’s t test ***P < 0.000 1, **P < 0.001.

To investigate the biological function of AtUBL5a and AtUBL5b, we generated transgenic plants expressing RNAi constructs for full-length *AtUBL5b* to reduce the expression of *AtUBL5* genes. We isolated two independent plant lines, namely RNAiUBL5b#1 and RNAiUBL5b#2. These plants had severe defects in root elongation and development, as well as reduced lateral root formation in response to auxin compared to that of wild-type plants (WT) (Fig 2A, 2B and 2E). The mRNA levels of both *AtUBL5a* and *AtUBL5b* decreased in RNAiUBL5b plants (Fig 2C).

There is a functional relationship between root development and the auxin response. Therefore, we investigated the response of RNAiUBL5b plants to auxin treatment by assessing lateral root development. RNAiUBL5b plants had a reduced response to auxin compared to that of WT plants (Fig 2D). We used single T-DNA insertion to disrupt *AtUBL5a* and *AtUBL5b* in *A*. *thaliana* (S1A Fig), but the T-DNA insert did not result in abnormal plant development (S1E and S1F Fig). The results of qRT-PCR using these single mutants indicated that *AtUBL5b* expression level was not downregulated by T-DNA (S1B Fig). Because T-DNA was inserted into the promoter region of *AtUBL5b* and the coding region of *AtUBL5a*, both genes were still expressed (S1C Fig). Thus, the *AtUBL5aAtUBL5b* double T-DNA insertion mutant might not be a null mutant, and may still grow normally (S1E and S1F Fig). The T-DNA insertion in *AtUBL5a* was located after the Asp22 residue, so that the *AtUBL5b* T-DNA insertion mutant generated a truncated AtUBL5b protein, which might be functional. Using RNAi instead to generate RNAiUBL5b plants resulted in knockdown of both *AtUBL5a* and *AtUBL5b*, although the off-target effects of RNAi should not be ignored. Knockout of Hub1 reduces cell viability in human cells [8]. Similarly, AtUBL5 is important for plant development. The double knockout of *AtUBL5a* and *AtUBL5b* genes might be lethal in *A*. *thaliana*, as it is in human cells. Further analysis of *A*. *thaliana* knockout mutants would illuminate this matter.

**Fig 3:**
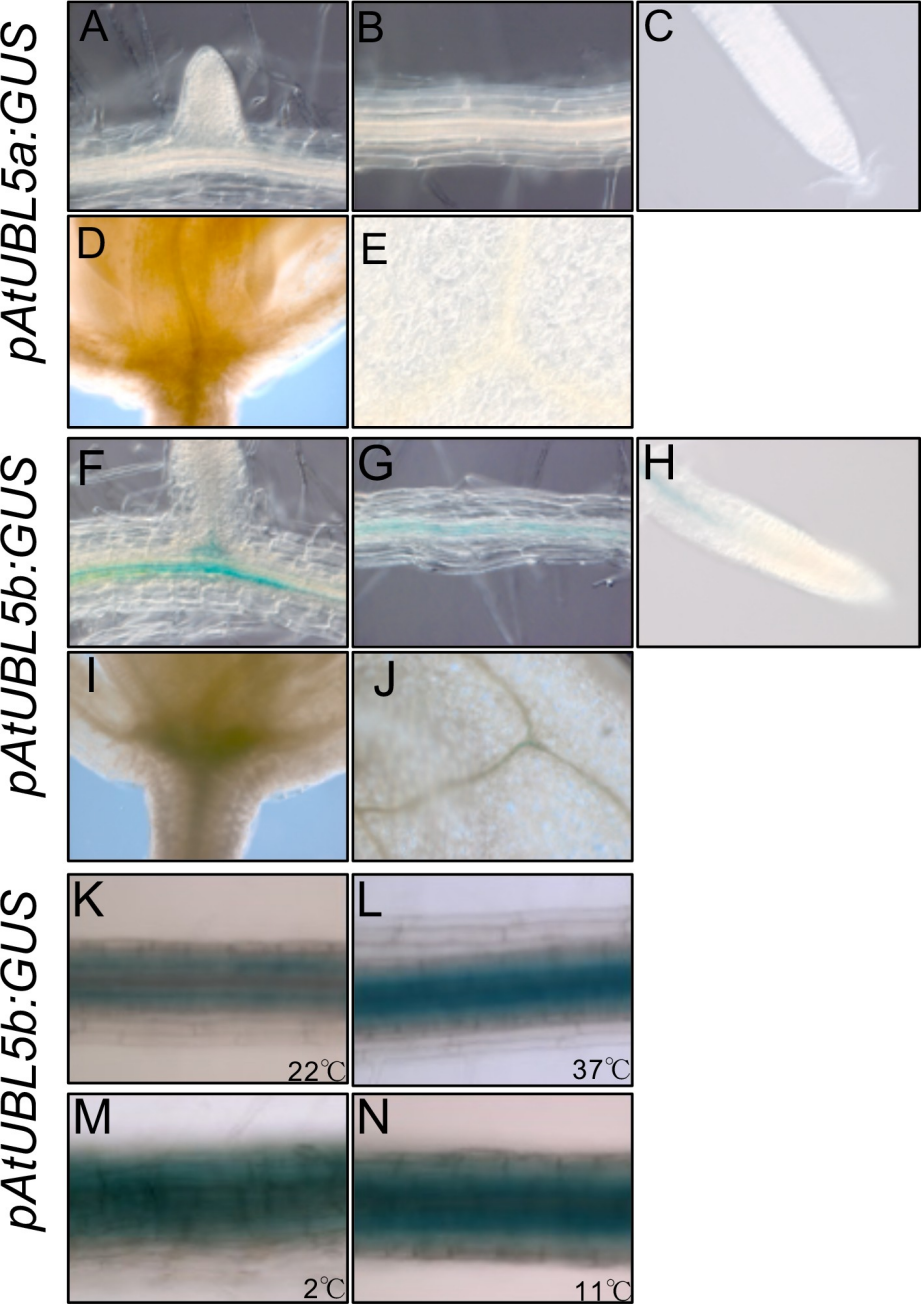
Histochemical analysis of *AtUBL5a* and *AtUBL5b* expression. (A-E) GUS staining in *pAtUBL5a*:*GU*S plant tissue. (F-J) GUS activity in *pAtUBL5b*:*GUS* plant tissue. Emerging LR (lateral root) primordium (A and F), primary root (B and G), primary root apex (C and H), primordial vein (D and I), and leaf vein (E and J). (K-N) GUS staining in *pAtUBL5b*:*GUS* plants at 22°C, K, or under heat stress (37°C, L) and cold stress (2°C and 11°C, M and N).

To understand the tissue specificity of *AtUBL5a* and *AtUBL5b* expression, we generated *A*. *thaliana* plants expressing β-glucuronidase (*GUS*) gene downstream of the *AtUBL5a* promoter, designated *pAtUBL5a*:*GUS* (Fig 3A–3E), and the *AtUBL5b* promoter, designated *pAtUBL5b*:*GUS* (Fig 3F–3J). β-glucuronidase staining indicated that *AtUBL5b* is expressed at the lateral root initiation site (Fig 3F), in the primary root (Fig 3G and 3H), and in the primordial (Fig 3I) and leaf veins (Fig 3J), whereas GUS staining in plants expressing GUS-fused *AtUBL5a* did not reveal any β-glucuronidase activity in seedling tissues (Fig 3A–3E). Taken together, these findings indicate that AtUBL5b plays a key role in plant root elongation and development.

According to histochemical analysis, *AtUBL5b* expression increased in response to heat stress (37°C), (Fig 3L) compared to that of plants under control conditions at 22°C (Fig 3K). *AtUBL5b* expression was also higher under cold stress (2°C and 11°C) than in plants maintained at 22°C (Fig 3M and 3N). These results are consistent with the expression analysis using qRT-PCR (S1D Fig). These results suggest that AtUBL5b might be involved in the regulation of cellular processes and the response to abiotic stresses, such as heat and cold, to adapt to the environment. In *L*. *perenne*, the overexpression of LpHub1 results in an improved stress response according to cell viability assessment [14]. In *A*. *thaliana*, AtUBL5 might have a role in stress response that is similar to that of LpHub1.

### Both AtUBL5a and AtUBL5b interact with the C-terminal domain of AtPRP38 protein containing the HIND

**Fig 4:**
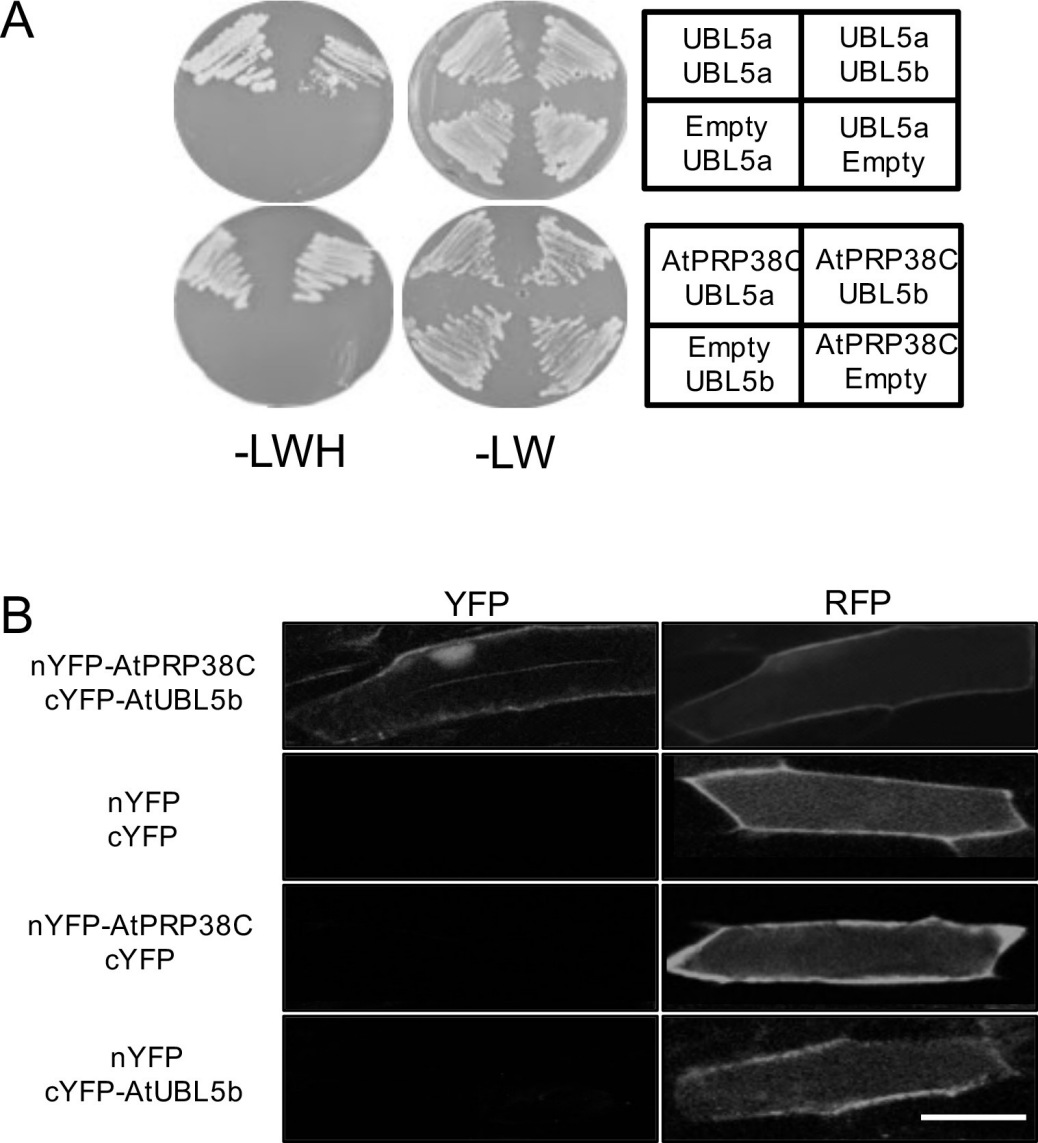
AtUBL5a and AtUBL5b interact with the C-terminal domain of AtPRP38. (A) Interactions between AtUBL5a, AtUBL5b, and AtPRP38C were detected using a yeast two-hybrid system. The AH109 strain was co-transformed with the constructs indicated, carrying a binding domain and an activation domain, and grown on synthetic drop-out (SD) media lacking leucine and tryptophan (LW) or leucine, tryptophan, and histidine (LWH) with 0.5 mM 3-amino-1,2,4-triazole (3-AT). Yeast containing both vectors could grow on SD-LW. Positive interactions appear as white spots on SD-LWH. (B) BiFC analysis of interactions between AtUBL5b and AtPRP38C. The cDNA constructs (left) were introduced into onion epidermis by particle bombardment, and fluorescence was observed after 1 day. YFP shows the reconstituted YFP fluorescence signal, showing the interaction between AtUBL5b and AtPRP38C in the nucleus. RFP indicates successful introduction of vectors. nYFP and cYFP denote the amino- and carboxyl-terminal halves of YFP, respectively. Bar = 50 μm.

In the present study, we used the yeast two-hybrid system to determine potential targets of AtUBL5. The C-terminal HIND of *A*. *thaliana* AtPRP38 interacts with yeast Hub1 through Asp22 non-covalently and yeast HUB1 binds to *Plasmodium falciparum* PRP38 according to an immunoprecipitation assay [5]. However, there is no evidence that *A*. *thaliana* UBL5 has a similar system [5]. To understand the direct interactions between AtPRP38C and AtUBL5, we performed yeast two-hybrid analysis to investigate the interaction between AtPRP38C containing the HIND and AtUBL5b, and found that AtPRP38C could bind to AtUBL5b (Fig 4A) indicating that AtUBL5b and AtPRP38 form a protein complex. AtPRP38 also has a HIND on its C-terminal domain with an Arg residue. In the *Arabidopsis* genome, there is no Snu66 gene homologue. Using the two-hybrid system, we also identified interaction between AtUBL5a and AtUBL5b (Fig 4A). As an alternative approach to verify the interaction between AtUBL5b and the AtPRP38C, we performed bimolecular fluorescence complementation (BiFC) assays *in vivo* (Fig 4B). YFP signal was detected in the nucleus when nYFP-AtPRP38C and cYFP-AtUBL5b (where nYFP and cYFP denote the amino- and carboxyl-terminal halves of YFP, respectively) were co-expressed in onion cells (1st row in Fig 4B), but not in negative control experiments (2nd row for nYFP and cYFP, 3rd row for cYFP and nYFP-AtPRP38C, and 4th row for nYFP and cYFP-AtUBL5b in Fig 4B). These results might indicate that AtUBL5b and AtPRP38C form a complex in plant cells. This suggests that AtUBL5b might work with AtPRP38 in the splicing complex in *Arabidopsis*, which is similar to the system in yeast and human cells. AtPRP38 has a HIND containing an Arg residue. Snu66 also has an Arg residue in its HIND that is involved in interactions with Hub1 [5]. Although the *Arabidopsis* genome contains no Snu66 gene homologue, the Arg residue in AtPRP38 would be involved in interactions with AtUBL5, like in other Hub1 interactions.

In the present study, we found that AtPRP38C interacts with AtUBL5a and AtUBL5b, and that AtUBL5a and AtUBL5b interact with each other, but there might be other unknown targets of AtUBL5a and AtUBL5b in *A*. *thaliana*. UBL5 proteins also have several highly conserved Lys residues, which could support the assemblage of ubiquitin-like chains. It is possible that AtUBL5 possesses the same properties as other ubiquitin-like modifiers, and be able to create ubiquitin-like chains. Yeast Hub1 interacts with Snu66 through the HIND, which stabilizes the processing complex in yeast and human cells, and is also conserved in AtPRP38C [5]. Similarly, AtUBL5b might work to stabilize the processing complex through the HIND of AtPRP38 in response to environmental responses in *A*. *thaliana*. Future identification of other AtUBL5 targets could reveal previously unexplored biological functions.

### Pre-mRNA splicing defects of *AtCDC2* in RNAiUBL5b

**Fig 5:**
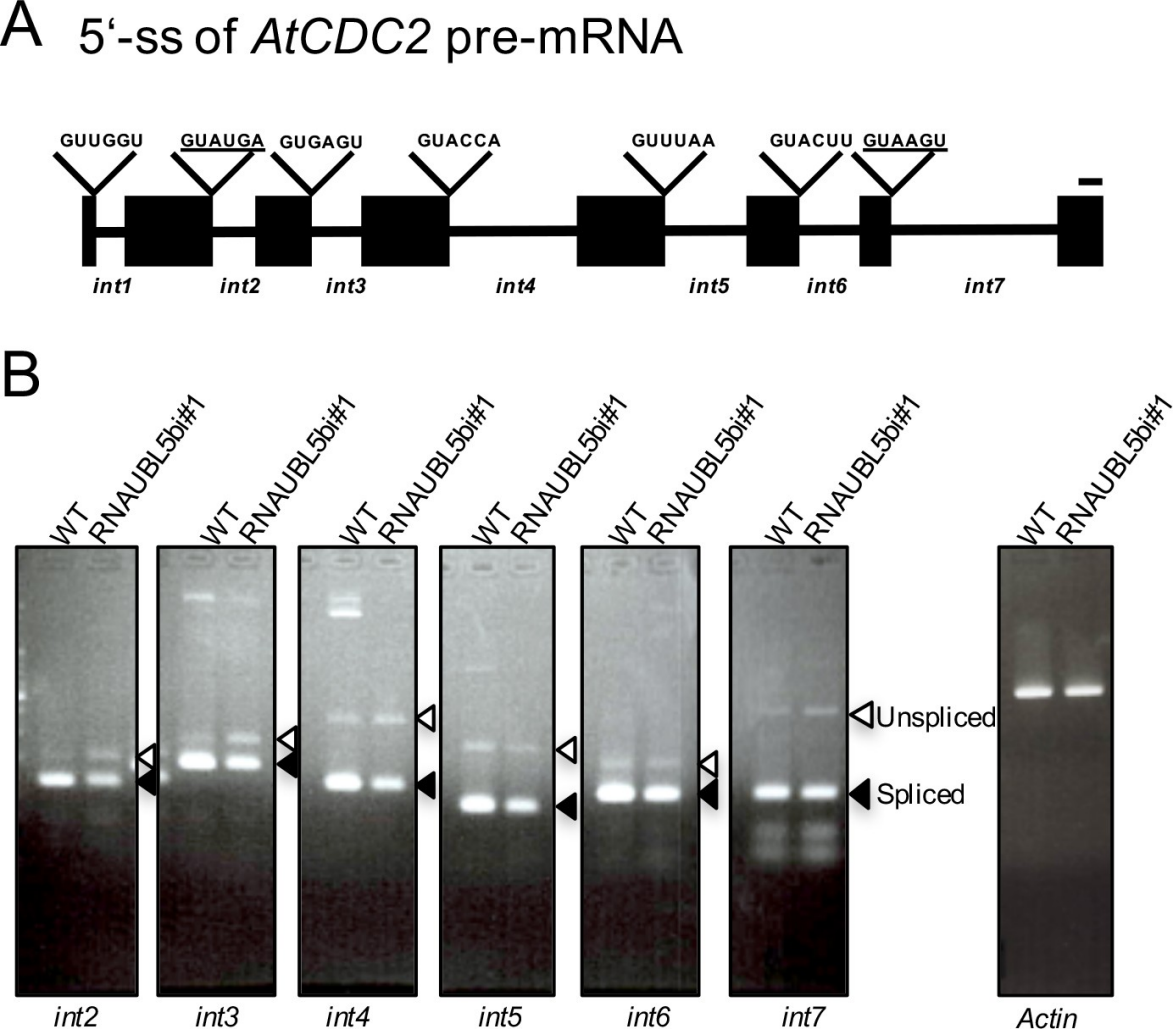
Pre-mRNA splicing of *AtCDC2* in RNAiUBL5b plants. (A) 5’ splicing site (5’-ss) of *AtCDC2* introns. Filled boxes indicate the exons of *AtCDC2*. Lines represent the introns of *AtCDC2*. Noncanonical 5’-ss sequences are underlined. Bar = 20 bp. (B) Pre-mRNA splicing defects of introns in *AtCDC2* were analyzed by qRT-PCR in RNAiUBL5b#1 and WT plants. Different pairs of *AtCDC2* primers were used to analyze the mRNA splicing defects at each intron. Filled and open arrowheads indicate the spliced and unspliced forms of PCR products, respectively. Primers were designed to detect both spliced and unspliced versions of each gene at each site.

To our knowledge, the role of AtUBL5 interacting with other proteins in plants has not been previously published. In the present study, we have demonstrated that AtUBL5 might have a specific role in the response to abiotic stress or phytohormones in plants. We hypothesize that AtUBL5b affects plant development through regulating pre-mRNA splicing of target genes. Therefore, it is possible that key genes in root development are not correctly spliced in RNAiUBL5b plants. Previous reports have identified a cyclin dependent kinase Cdk1/Cdc2 gene, *Cdc2*, as a pre-mRNA splicing target of yeast Hub1 [6]. We therefore performed qRT-PCR to investigate the splicing pattern of *A*. *thaliana* Cdc2 (*AtCDC2*) (Fig 5A), and found that *intron2*, *intron3*, and *intron4* of *AtCDC2* were not spliced correctly in RNAiUBL5b compared to in WT plants (Fig 5B). These results are similar to those of yeast *Hub1* knockout [5]. *Intron 5* and *intron 6* of *AtCDC2* which are canonical sequences were spliced properly in RNAiUBL5b compared to in WT plants. *Intron 7* of *AtCDC2* is not also spliced normally. The splicing site of *intron7* in *AtCDC2* is a noncanonical splicing site, which is also independently processed by Hub1 in *S*. *cerevisiae* [5]. Further study on these splicing difference in *A*.*thaliana* by knock down of *AtUBL5* would be studied more detail in future.

**Fig 6:**
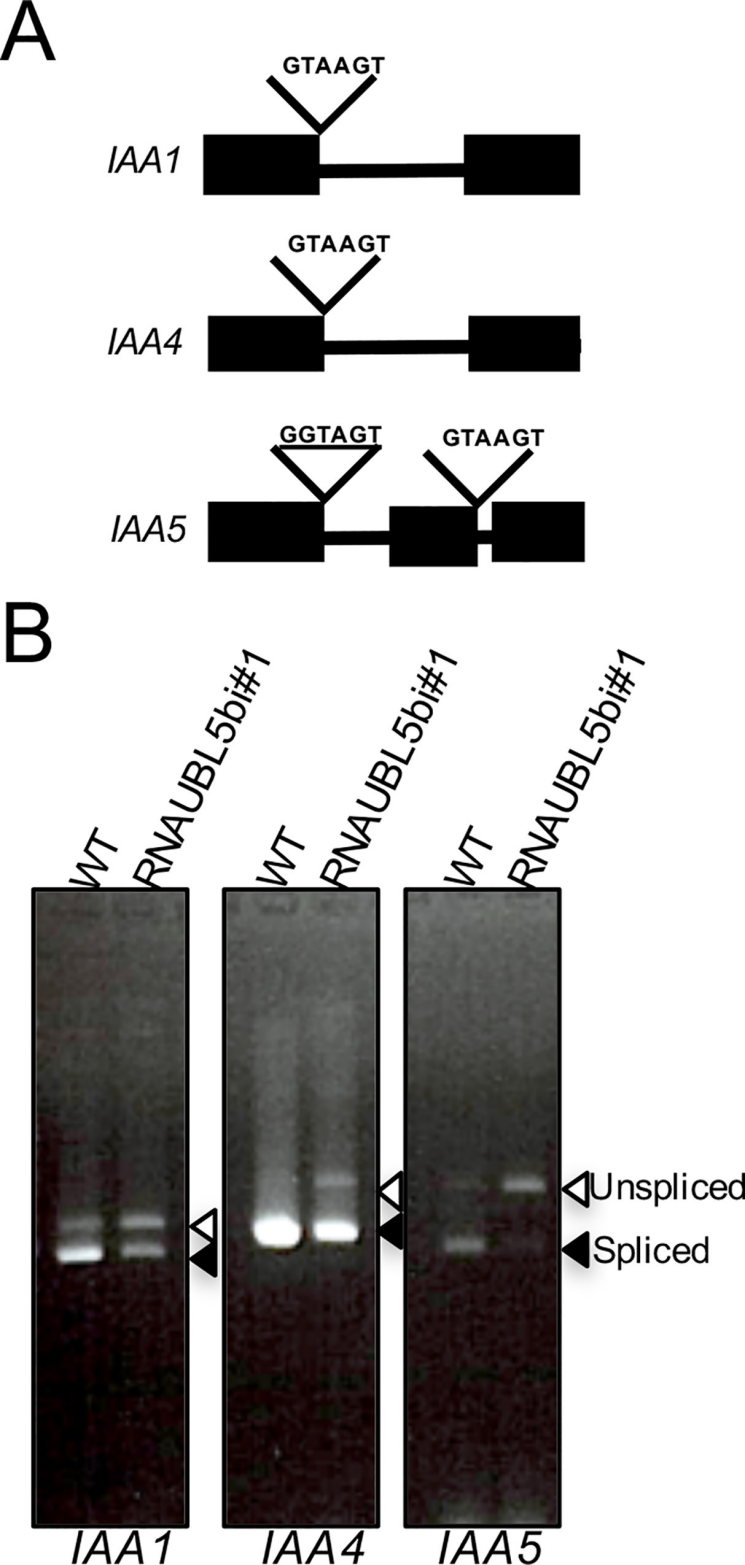
Pre-mRNA splicing of *IAA* genes in RNAiUBL5b and WT. (A) 5’-ss of *IAA1*, *IAA4*, and *IAA5* introns. The noncanonical 5’-ss sequence is underlined. (B) Splicing defects of the introns in the *IAA* genes were assessed by qRT-PCR, comparing RNAiUBL5b#1 with WT plants. Filled and open arrowheads indicate the spliced and unspliced forms, respectively.

We observed phenotypic defects in root elongation, and developmental abnormalities in response to auxin in RNAiAtUBL5b plants (Fig 2D and 2E). We therefore also assessed the pre-mRNA splicing of the introns in indole acetic acid (IAA) family genes *IAA1*, *IAA4*, and *IAA5*. The 5’ -ss of introns in *IAA1* and *IAA4* are typical splicing sites, whereas *IAA5* has both noncanonical and canonical splicing sites. Among the three *IAA* genes tested, the correct splicing of *IAA5* was the most affected in RNAiUBL5b plants. We found that abnormally spliced pre-mRNA products of IAA1 and IAA4 slightly accumulated in RNAiUBL5b plants compared to in the WT plants (Fig 6A and 6B). This effect may be explained by the fact that the splice site sequence in *IAA5* is different from that in *IAA1* and *IAA4*, which share the same sequence (Fig 6A). This suggests that AtUBL5b is involved in pre-mRNA splicing of genes required for the response to auxin through modulation of AtPRP38 activity. Therefore, we also tested for splicing defects in *AtPRP38* and found that abnormal pre-mRNA splicing products also accumulated in these plants (S2 Fig). These findings indicate that key genes involved in root elongation and lateral root development would be spliced via the spliceosome containing AtPRP38 and modulated by AtUBL5b.

We next examined the complex regulation processes of AtUBL5 targets, which revealed previously unknown putative AtUBL5 functions in *A*. *thaliana*. Our results suggest that AtUBL5 regulates the pre-mRNA splicing of *AtCDC2* at a noncanonical splicing site, and a similar mechanism is also observed in yeast systems. AtUBL5 also regulates splicing at canonical sequences, which is also similar to yeast Hub1. In *S*. *cerevisiae*, pre-mRNA splicing defects were observed in noncanonical sequences, but not for canonical splicing sequences [5]. In contrast, knockout of Hub1 in *S*. *pombe* induced splicing defects in both noncanonical and canonical sequences, similar to what we observed in *A*. *thaliana*. In human cells, there are no specific splicing sites recognized by Hub1, which modulates spliceosome activity [2]. There is no similarity between the splice sites sensitive to human Hub1 [2], leading to different patterns of splicing sites among organisms. According to microarray analysis, human Hub1 stimulates RNA processing by stabilizing the pre-mRNA splicing complex, which contains Snu66, and thus affects a wide variety of splicing events. These events are associated with different transcripts and lead to alternative splicing of target pre-mRNAs, which are identified by their characteristic recognition domains [2]. Thus, there would be a unique selection mechanism for splicing sites in each biological system.

## Conclusions

**Fig 7:**
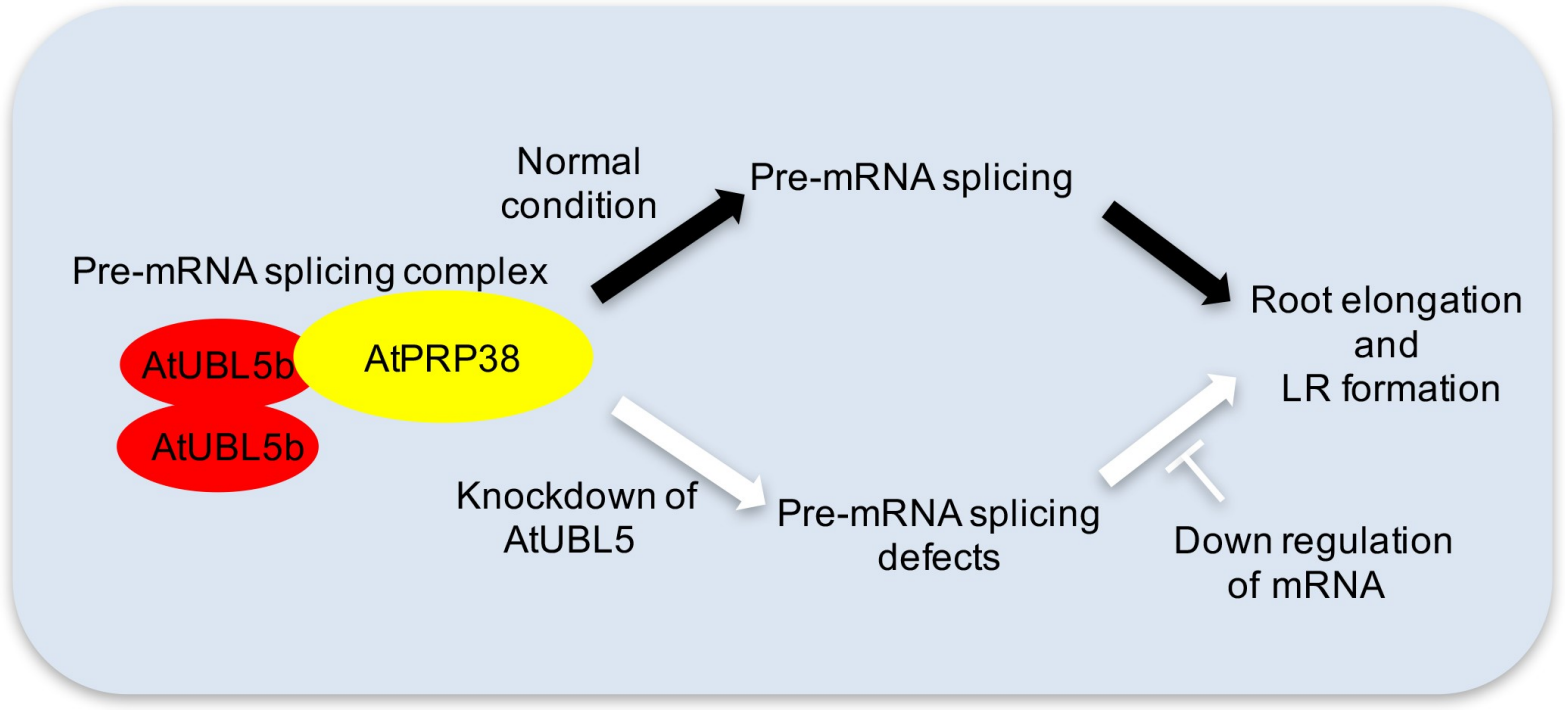
Schematic model of AtUBL5 function in pre-mRNA splicing in *A*. *thaliana*.

To the best of our knowledge, this is the first study to report that AtUBL5 is important for plant growth and development by regulating pre-mRNA splicing at both canonical and noncanonical splice site sequences in *A*. *thaliana*. The knockdown of AtUBL5 caused developmental defects in root elongation and development (Fig 7). We also showed that AtUBL5 and AtPRP38 interact in a highly conserved manner via the AtPRP38 C-terminal domain, which contains the HIND. Thus, AtUBL5 plays a highly conserved regulatory role in general pre-mRNA splicing in *A*. *thaliana* by modulating AtPRP38 activity in the splicing complex. This mechanism is similar to that observed in other model systems, such as yeast and human cells (Fig 7). We also revealed that AtUBL5 regulates root elongation and lateral root development in response to auxin.

In *A*. *thaliana*, knockdown of *AtUBL5* gene expression resulted in defects in lateral root development and primary root growth, indicating that AtUBL5 regulates root elongation and development in higher plants. AtUBL5b interacts with AtPRP38C, which contains the HIND. AtUBL5b concomitantly interacts with AtUBL5a. This AtUBL5-AtPRP38C interaction is critical for proper pre-mRNA splicing in root development, including regulation of the expression of responsive genes.

## Materials and methods

### Bioinformatics and phylogenetic analysis

To identify *A*. *thaliana* Hub1 homologues, we performed a Basic Local Alignment Search Tool (BLASTp) search with *S*. *cerevisiae* Hub1 protein sequence within the *A*. *thaliana* genome. Two Hub1 homologues were identified with significant sequence identity to yeast Hub1 in the *A*. *thaliana* translated genome database (ATH1_seq database) accessed from TAIR (The *Arabidopsis* Information Resource). A reciprocal search with AtUBL5 sequences found that *S*. *cerevisiae* Hub1 had the highest sequence similarity in the database and confirmed the orthologous relationship between these proteins. A BLAST search with yeast *Hub1* was used to screen DNA sequences for candidate plant *HUB1* homologues in the nucleotide collection database. We manually analyzed the data by removing incomplete sequences, removing possible redundant sequences, and removing unrelated sequences. The selected sequences were analyzed using MEGA7 software. The Multiple Sequence Comparison by Log-Expectation (MUSCLE) program was used to generate multiple sequence alignments. A phylogenetic tree was constructed using the Neighbor-Joining method. Yeast *Hub1* was used as an out-group parameter in the phylogenetic analysis. The percentage of tree reproducibility was calculated with bootstrap testing using 500 replicates. Interactive tree of life (iTOL, https://itol.embl.de) and Adobe Illustrator were used for graphic visualization of the tree. The amino acid sequences of Hub1 from selected species were collected from the non-redundant protein database using BLAST.

### Plant materials and growth conditions

All *A*. *thaliana* plants used in this study were the Col-0 ecotype. Plant transformation vectors were introduced into Agrobacterium tumefaciens strain by electroporation [19]. Transformed Agrobacterium cells were spread onto an LB agar plate containing kanamycin (Sigma, St. Louis, MO) or spectinomycin (Sigma). Positive transformants were identified by performing sequence-specific colony PCR. *Arabidopsis* were transformed by infiltration [20]. Seeds from infiltrated plants were sterilized using a bleach solution and selected on standard MS medium (0.5 × Murashige and Skoog salts and 1.3 mM 2-(4-morpholino)ethanesulfonic acid (MES)-KOH (pH 5.7), with the addition 0.7% (w/v) phytoagar for solid medium) containing the appropriate inhibitors: 50 mg/L kanamycin (Sigma), 10 mg/L glufosinate ammonium (Crescent Chemical Co. Islandia, NY), and/or 25 mg/L hygromycin (Sigma) with 100 mg/L cefotaxime (Sigma). The T_1_ generation that were resistant to the appropriate inhibitors were collected from T_0_ transformants. T_2_ generation that were fully resistant homozygotes to the appropriate inhibitors were collected from T_1_ generation. At least two independent lines of the T_2_ generation plants were used in subsequent experiments.

Plants were grown on MS medium containing 1% (w/v) sucrose under long day conditions (16 h light and 8 h dark). For phenotypic analysis, plants were grown on half-strength MS media with 1% (w/v) sucrose and 1% (w/v) agar for 10 days. To study the response to auxin, plants were grown on half-strength MS media for 5 days, and then transferred onto 2,4-dichlorophenoxyacetic acid (2,4-D; Sigma)-containing plates. The concentration of 2,4-D in each plate was 0, 50, 100, and 200 nM. Then, after 6 days, Plants were photographed and analyzed with ImageJ software version 1.50j and the density of emerged lateral roots and elongation of primary roots were examined.

To analyze the response to temperature, plants were grown on solid MS medium for 15 days at 22°C, and then transferred to 37°C, 2°C, or 11°C for 12 hours. Each plant was used for this study.

All T-DNA insertion *Arabidopsis* lines were either described previously or obtained as T-DNA insertions from the *Arabidopsis* Biological Resources Center (ABRC) at Ohio State University [21]. *Arabidopsis* T-DNA insertion lines of *AtUBL5a* (SALK_126377C), *AtUBL5b* (SALK_024051C), and *AtPRP38* (SALK_049269C and SALK_021778C) were obtained from ABRC (S1A Fig). T-DNA insertion sites were confirmed by sequencing of PCR-amplified fragments using LBb1 primer and specific primers.

### Statistical analysis

All experiments in this study were repeated at least three times. Groups were compared using Student's *t* test. Asterisks indicate statistically significant differences (***P < 0.000 1, **P < 0.001).

### *A*. *thaliana UBL5* sequences and molecular cloning

The DNA sequences encoding *AtUBL5*a (At3g45180), and *AtUBL5b* (At5g42300) were amplified by PCR using specific primers pairs based on the genome of *A*. *thaliana* (S1 Table). PCR products were then cloned using the pENTR^™^/D-TOPO^™^ Cloning kit (Thermo Fisher Scientific, Waltham, MA). The vectors containing *AtUBL5*a or *AtUBL5b* were used for recombination in Gateway^™^ vectors using LR clonase^™^ (Thermo Fisher Scientific). These *AtUBL5a and AtUBL5b* genes were also transferred to a Gateway vector, pUGW6 (a gift from T. Nakagawa) [22], to yield pUGW6-AtUBL5a and pUGW6-AtUBL5b, which express GFP fusion proteins under a 35S promotor. The pUGW6-AtUBL5a and pUGW6-AtUBL5b plasmids were used for particle bombardment experiments.

To generate constructs for GUS staining using *AtUBL5*a or *AtUBL5b* promoters, genomic fragments were used in PCR assays to amplify the promoter region of *AtUBL5a* (1,500 bp) and *AtUBL5b* (1,500 bp) using specific primers (S1 Table). The amplified regions were cloned into a pENTR^™^/D-TOPO plasmid (https://www.addgene.org/vector-database/2519/) to obtain pENTR-AtUBL5a. The *AtUBL5a* promoter region was transferred to a Gateway binary vector, pGWB203 (a gift from T. Nakagawa) [22], to yield a pGWB203-AtUBL5a promoter using LR clonase^™^ (Thermo Fisher Scientific). The AtUBL5b promoter sequence upstream of the ATG start codon (from -1,500 bp to +3 bp) was amplified by PCR, cloned into the pBluescript SK (-) vector (https://www.addgene.org/vector-database/1947/) and then cloned into the *Hin*d III and *Bam* HI sites of the pBI101 vector (Clontech, Palo Alto, CA, USA).

For RNAi vector construction, the full-length cDNA of pENTR-AtUBL5a and pENTR-AtUBL5b were transferred to a Gateway vector, pH7GWIWG2(I) (Functional Genomics, Division of the Department of Plant Systems Biology [VIB, the Flanders Institute for Biotechnology, Ghent University]) to yield pH7GWIWG2-RNAiAtUBL5a and pH7GWIWG2-RNAiAtUBL5b using LR clonase^™^ (Thermo Fisher Scientific). The resulted vectors express RNAi constructs for full-length cDNA of AtUBL5b under the control of 35S promoter.

For two-hybrid analysis, *AtUBL5a and AtUBL5b* genes were also transferred to Gateway vectors, pGBD-C1-GW [23] and pGAD-C1-GW [23], to yield pGBD-C1-GW-AtUBL5a, pGBD-C1-GW-AtUBL5b, pGAD-C1-GW-AtUBL5a and pGAD-C1-GW-AtUBL5b, respectively, using LR clonase^™^ (Thermo Fisher Scientific). A modified *AtPRP38C* (see BiFC section) was also transferred to a Gateway vector, pGAD-C1-GW2, to yield pGAD-C1-GW2-AtPRP38C using LR clonase^™^ (Thermo Fisher Scientific).

### Bimolecular fluorescence complementation (BiFC) assay

To construct fusion genes for the BiFC assay, the entire coding region of AtUBL5b was transferred from pENTR-AtUBL5b into the Gateway expression vector pnYGW (a gift from T. Nakagawa) [24] to yield pNYFP-AtUBL5b using LR clonase^™^ (Thermo Fisher Scientific). The resultant construct encodes a fusion protein consisting of the amino-terminal half of YFP fused to the amino-terminal portion of AtUBL5b. cDNA encoding *AtPRP38* was obtained by RT-PCR from *A*. *thaliana*. The *AtPRP38* cDNA (1,065 bp) was amplified with KOD FX Neo DNA polymerase (Toyobo, Osaka, Japan) using the AtPRP38 primer set (S1 Table), and then introduced to the Gateway entry vector pENTR^™^/D-TOPO (Thermo Fisher Scientific) to yield pENTR-AtPRP38. The C-terminal region of *AtPRP38* cDNA as amplified with KOD FX Neo DNA polymerase (Toyobo) using the AtPRP38C primer set (S1 Table), and then introduced to the Gateway entry vector pENTR^™^/D-TOPO (Thermo Fisher Scientific) to yield pENTR-AtPRP38C. The *AtPRP38C* DNA fragment was transferred into the Gateway expression vector pcYGW (a gift from T. Nakagawa) [24, 25] to yield pCYFP-AtPRP38C using LR clonase^™^ (Thermo Fisher Scientific). These plasmids were used for particle bombardment experiments to perform the BiFC experiment. For BiFC analysis, YFP and RFP fluorescent signals in the epidermis were analyzed under an epifluorescence microscope (AxioImager Z1, Carl Zeiss) equipped with a CCD camera (AxioCam HRc, Carl Zeiss). RFP was also used to identify the cells in which the vectors were successfully introduced.

### Subcellular localization analysis

GFP fluorescent proteins were fused to the N-terminus of AtUBL5a and AtUBL5b to investigate the localization of GFP-AtUBL5a and GFP-AtUBL5b, respectively. Gold particles (1.0 μm; Bio-Rad, Richmond, CA) coated with DNA (0.1 μg/μL) were delivered into onion epidermal cells using the Biolistic^®^ PDS-1000/He system (Bio-Rad, Richmond, CA) with 1,100 psi rupture discs. After incubation at 22°C for 16 hours, GFP fluorescent signals in the onion epidermal cells were recorded under an epifluorescence microscope (AxioImager Z1, Carl Zeiss) equipped with a CCD camera (AxioCam HRc, Carl Zeiss). The empty vector pA7-GFP was used a as control. Images were processed using Spot Advance and Adobe Photoshop software.

### GUS analysis

Transgenic plants harboring pAtUBL5a-GUS and pAtUBL5b-GUS were stained for GUS activity as previously described [26]. Plasmids were inserted into *Agrobacterium tumefaciens* strain EHA105, and then transformed into *Arabidopsis* using the floral dip method as described previously [20]. Tissues from pAtUBL5a-GUS and pAtUBL5b-GUS-transformed plants were immersed in GUS solution [1 mM X-gluc, 100 mM sodium phosphate buffer (pH 7.0), 0.5 mM K_3_Fe(CN)_6_, 0.5 mM K_4_Fe(CN)_6_, 10 mM EDTA, and 0.1% (v/v) Triton X-100] and incubated for 12 h at 37°C. Ethanol was used to remove the chlorophyll. Stained plants were observed under an Axioskop microscope (Carl Zeiss) coupled to an Insight digital camera.

### Yeast two-hybrid analysis

Yeast two-hybrid analysis was performed using two plasmids that were co-transformed into yeast strain AH109 according to the Matchmaker^®^ GAL4 Two-hybrid System 3 instructions (Takara Bio Inc., Shiga, Japan). Transformed yeast was selected on SD-LW (synthetic drop-out media lacking leucine and tryptophan). Interactions were tested on selective media lacking leucine, tryptophan, adenine, and histidine (SD-LWH), with 0.5 mM 3-AT according to the manufacturer’s instructions.

### RNA isolation, cDNA synthesis, and quantitative polymerase chain reaction

RNA was extracted from leaf and root tissue using QIAGEN RNeasy Plant Mini Kit according to the manufacturer’s instructions (Qiagen, Hilden, Germany). cDNA was synthesized with illustra^™^ Ready-to-Go^™^ RT-PCR Beads (GE Healthcare, Buckinghamshire, UK). Quantitative RT-PCR was performed with SYBR^®^ premix PCR mix (Takara Bio Inc.) using a GeneAmp PCR system 9077 (Applied Biosystems, Waltham, CA). Primer sets are listed in S1 Table.

## Supporting information

S1 Fig T-DNA insertion lines of *AtUBL5a* and *AtUBL5b*. (A) Map of T-DNA insertion sites in *AtUBL5a* and *AtUBL5b* mutants of *A*. *thaliana*. Bar = 20 bp. Filled arrowheads indicate the site of T-DNA insertion. The black arrows indicate the primers used for qRT-PCR. (B) qRT-PCR of *AtUBL5a* and *AtUBL5b*. The expression level of each gene was normalized to that of *ACT2*. Error bars denote the SE of three independent biological replicates. (C) qRT-PCR of *AtUBL5a* and *AtUBL5b*. The expression level of each gene was normalized to that of *ACT2*. Error bars denote the SE of three independent biological replicates. (D) qRT-PCR of *AtUBL5b* in response to different temperatures (37, 2, 11, or 22°C). The expression level of *AtUBL5b* was normalized to that of *ACT2*. Error bars denote the SE of three independent biological replicates in (B, C, D). (E) Growth of *atubl5aatubl5b* and WT plants. Plants were grown for 11 days. Bar = 10 mm. (F) Root length of *atubl5aatubl5b* and WT plants. Values in (F) represent mean ± standard deviation of 8–10 samples in three independent experiments.
(TIF)

S2 Fig T-DNA insertion in *AtPRP38* results in pre-mRNA splicing defects. (A) Map of T-DNA insertion sites in *AtPRP38* mutants. The gray box indicates the HIND of AtPRP38. Bar = 20 bp. (B) Pre-mRNA splicing of the *IAA1* gene in the *AtPRP38* mutant.
(TIF)

S1 Table Sequences of oligonucleotide primers used in the study. (TIF)
