# Supplementary figures and images for: AtUBL5 regulates growth and development through pre-mRNA splicing in Arabidopsis thaliana
Source: PLoS One. 2019 Nov 15;14(11):e0224795. doi: 10.1371/journal.pone.0224795 (PMC6857937; doi:10.1371/journal.pone.0224795)

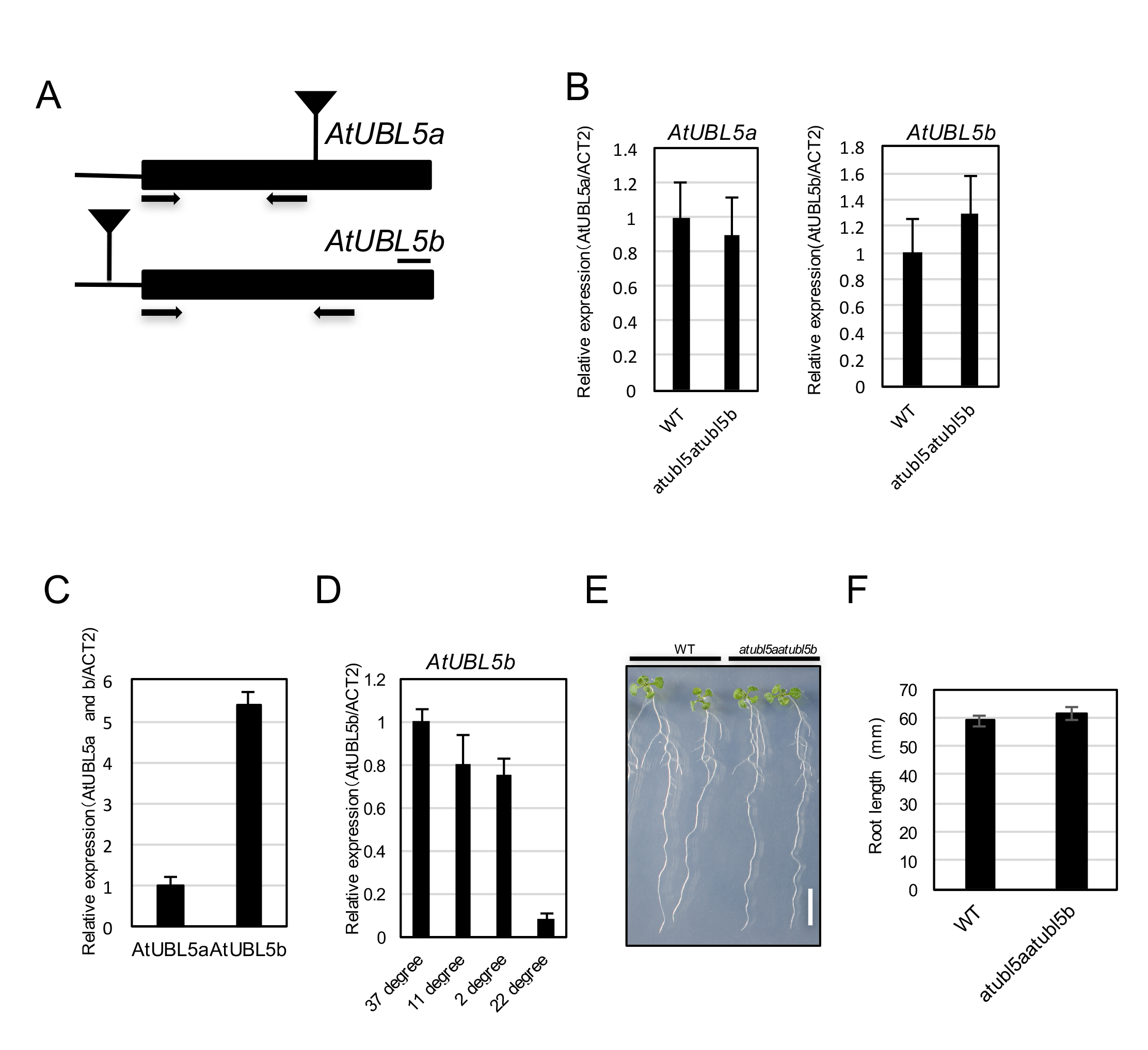

Supplement: S1 Fig — (A) Map of T-DNA insertion sites in AtUBL5a and AtUBL5b mutants of A. thaliana. Bar = 20 bp. Filled arrowheads indicate the site of T-DNA insertion. The black arrows indicate the primers used for qRT-PCR. (B) qRT-PCR of AtUBL5a and AtUBL5b. The expression level of each gene was normalized to that of ACT2. Error bars denote the SE of three independent biological replicates. (C) qRT-PCR of AtUBL5a and AtUBL5b. The expression level of each gene was normalized to that of ACT2. Error bars denote the SE of three independent biological replicates. (D) qRT-PCR of AtUBL5b in response to different temperatures (37, 2, 11, or 22°C). The expression level of AtUBL5b was normalized to that of ACT2. Error bars denote the SE of three independent biological replicates in (B, C, D). (E) Growth of atubl5aatubl5b and WT plants. Plants were grown for 11 days. Bar = 10 mm. (F) Root length of atubl5aatubl5b and WT plants. Values in (F) represent mean ± standard deviation of 8–10 samples in three independent experiments. (TIF) [file pone.0224795.s001.tif]

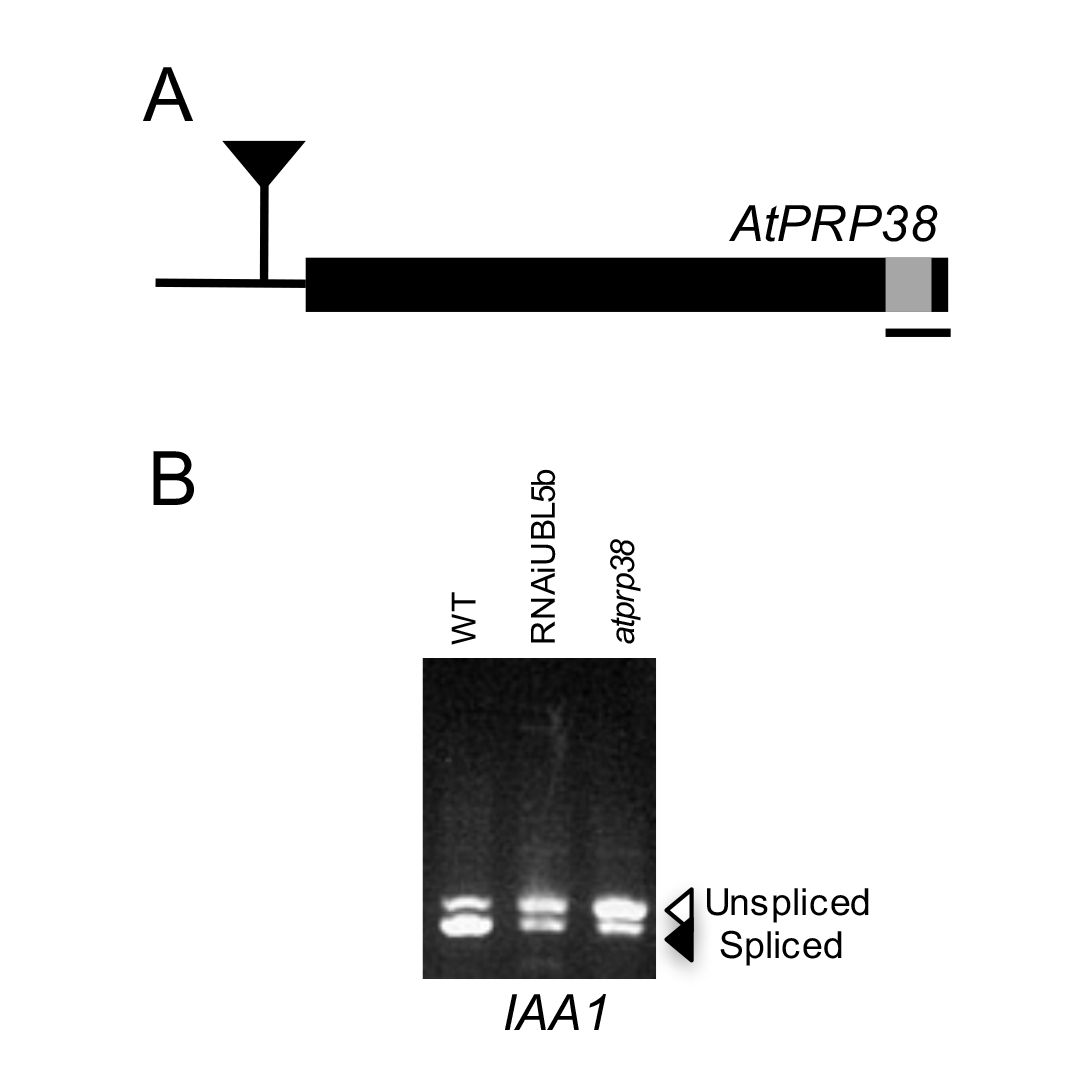

Supplement: S2 Fig — (A) Map of T-DNA insertion sites in AtPRP38 mutants. The gray box indicates the HIND of AtPRP38. Bar = 20 bp. (B) Pre-mRNA splicing of the IAA1 gene in the AtPRP38 mutant. (TIF) [file pone.0224795.s002.tif]

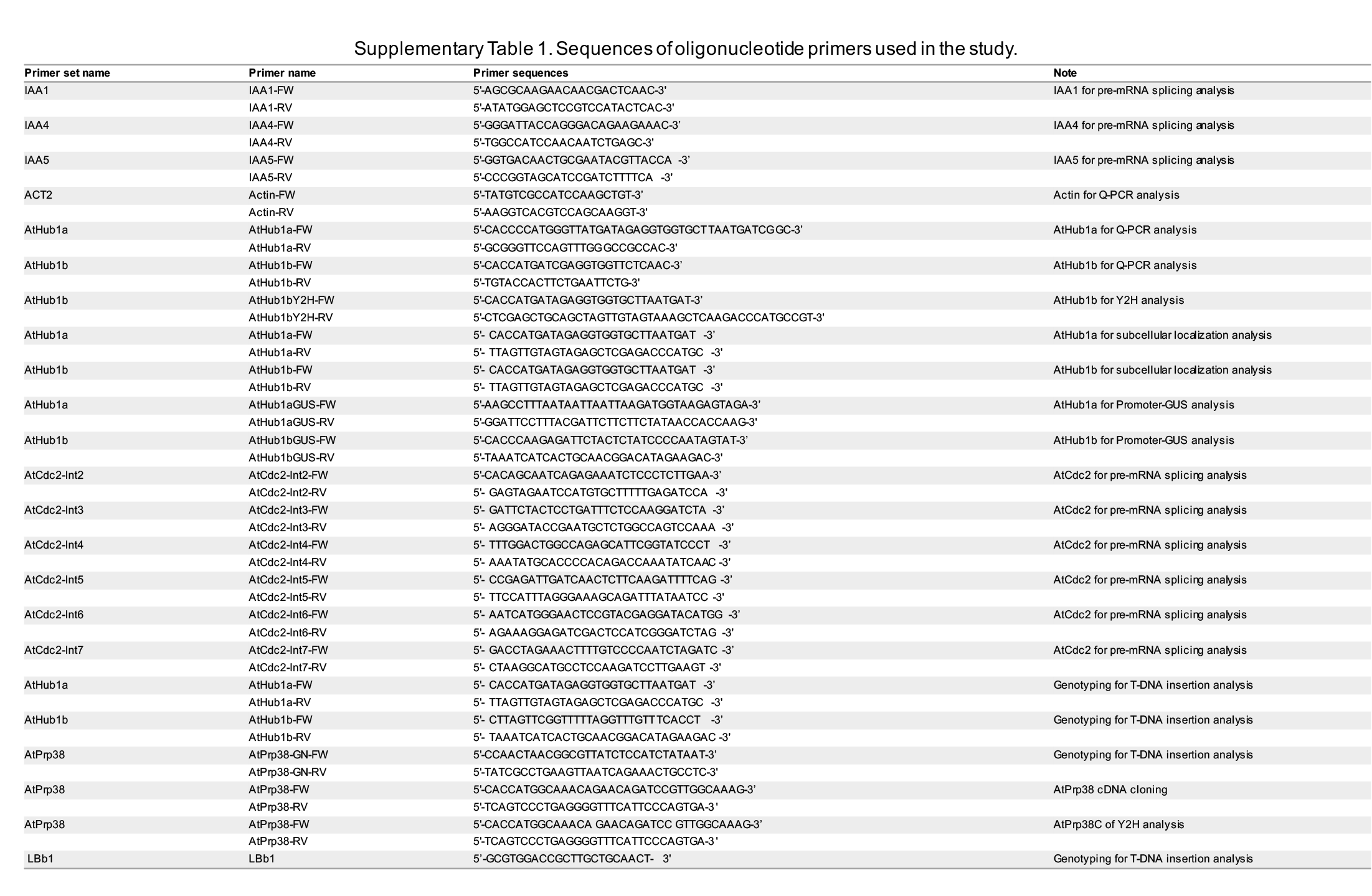

Supplement: S1 Table — (TIF) [file pone.0224795.s003.tif]
